# Thermal Behavior of Cylindrical Buckling Restrained Braces at Elevated Temperatures

**DOI:** 10.1155/2014/672629

**Published:** 2014-01-09

**Authors:** Elnaz Talebi, Mahmood Md. Tahir, Farshad Zahmatkesh, Airil Yasreen, Jahangir Mirza

**Affiliations:** ^1^UTM Construction Research Centre, Universiti Teknologi Malaysia (UTM), 81310 Johor Bahru, Johor, Malaysia; ^2^Robotics and Civil, Research Institute of Hydro-Quebec, Varennes, QC, Canada

## Abstract

The primary focus of this investigation was to analyze sequentially coupled nonlinear thermal stress, using a three-dimensional model. It was meant to shed light on the behavior of Buckling Restraint Brace (BRB) elements with circular cross section, at elevated temperature. Such bracing systems were comprised of a cylindrical steel core encased in a strong concrete-filled steel hollow casing. A debonding agent was rubbed on the core's surface to avoid shear stress transition to the restraining system. The numerical model was verified by the analytical solutions developed by the other researchers. Performance of BRB system under seismic loading at ambient temperature has been well documented. However, its performance in case of fire has yet to be explored. This study showed that the failure of brace may be attributed to material strength reduction and high compressive forces, both due to temperature rise. Furthermore, limiting temperatures in the linear behavior of steel casing and concrete in BRB element for both numerical and analytical simulations were about 196°C and 225°C, respectively. Finally it is concluded that the performance of BRB at elevated temperatures was the same as that seen at room temperature; that is, the steel core yields prior to the restraining system.

## 1. Introduction

The strength of buildings in hazardous loading conditions such as earthquake or fire is one of the main concerns for structural engineers. The main role of horizontal bracing systems is to sustain wind and earthquake loads on the structures. Because these systems append an additional strength which prevents the formation of progressive collapse in the structures, they can also be used to sustain forces that are generated in fire incidents. Previous studies [1] have shown that horizontal bracing systems have the efficacy of not only enhancing the lateral restraint of the structural frame, but also reducing the pullout of the columns, thus preventing the progressive collapse of the structures at elevated temperature [1].

The use of Buckling Restrained Brace systems (BRBs) has expanded in recent decades owing to their supreme structural behavior in earthquakes. Performance of BRBs under static and seismic loadings at ambient temperature [2–4] has been well studied, while the behavior of these systems at high temperatures has not yet been investigated to the best of authors' knowledge. Hence there is a dire need to investigate the thermal behavior of such braces at elevated temperatures.

**Figure 1:**
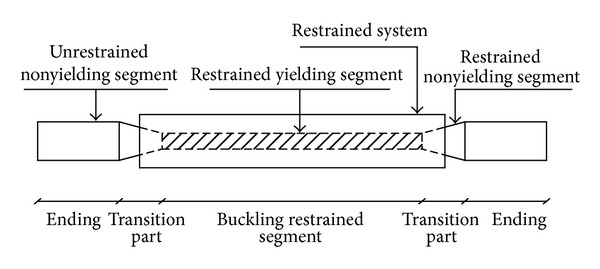
Buckling Restrained Brace (BRB) components [4].

The main role of Buckling Restrained Braced frames (BRBF) is the lateral resistance of structures in earthquakes. The principal specifications of these systems are high energy dissipation capability, high ductility, and almost symmetrical hysteretic responses both in tension and compression [4]. As shown in Figure 1, BRBs are composed of a yielding steel core encased in a concrete-filled steel hollow casing to prevent its buckling, nonyielding and buckling-restrained transition parts, and nonyielding and unrestrained end regions. About 60%–70% of the entire length of the core is restrained by the casing [4]. In these bracing systems, compression stresses are mainly sustained by the restrained portion of the core. On the other hand, the yield strength of the steel core is much lower than that of steel tube casing. It allows the core to yield in the same manner during tension and compression, prior to the casing, thus enhancing the energy dissipation capabilities of BRBs considerably in comparison to the ordinary bracing systems.

**Figure 2:**
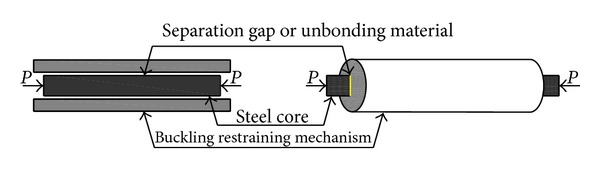
Buckling Restrained Brace system (BRBs) [5].

Due to Poisson's effect on the steel core, it expands when it is compressed. To prevent the axial stress transition from the core to the restrainer, a certain amount of clearance between the core and concrete must be provided to avoid the friction between them. To minimize the friction almost completely between the core and concrete, a debonding agent is applied to the surface of the core (Figure 2).

A further detailed history of BRBs can be seen elsewhere [6].

In this study, a three-dimensional nonlinear finite element model was developed to investigate the behavior of BRBs at elevated temperature. The nonlinear finite element analysis package ABAQUS [7] was utilized to perform the thermal and structural analysis. The thermal behavior of numerical model was validated by the analytical Green's function solution proposed by Wang and Tan [8]. On the other hand, the structural response was validated by simulation and comparison with the analytical model proposed by Choi and Xiao [9] and Choi [10]. Using validated model, the thermal responses of brace are shown which include the temperature-time history on the surfaces and different locations within the cross-section, as well as the structural response such as stress and strain histories of the specimen.

It is worth mentioning here that all of the notations used in this paper are described in the Notations Section.

## 2. Numerical FE Model

### 2.1. Process of the Analysis

As mentioned above, the finite element analysis software, ABAQUS [7], is utilized to represent the nonlinear behavior of BRBs beneath both thermal and structural actions. A sequentially-coupled thermal-stress analysis procedure in the software is used for the modeling. This technique was utilized since stress-displacement solutions are affiliated to the temperature history but there is no inverse dependency [7]. This approach consisted of two sequential analysis stages, where the outcome of first step was used to follow the analysis in the second step. These steps are as follows.

(*1) Heat Transfer Analysis*. It was carried out to simulate the heat transfer from the outer surface of the brace through its cross-section and along its length. 

(*2) Stress Analysis*. It was conducted in order to model the structural response of BRBs exposed to fire or thermal loading resulted in Step 1.

In order to transfer thermal analysis results to the structural analysis correctly, the type of elements must be identical in both steps. Furthermore, the time in both of the steps should be consistent with each other; only then, the temperature in thermal and structural analyses has the same meaning. Moreover, in order to prepare the condition in which data can be transmitted more effectively between the analyses, the finite element meshes should be the same in both steps.

### 2.2. Thermal Analysis

**Figure 3:**
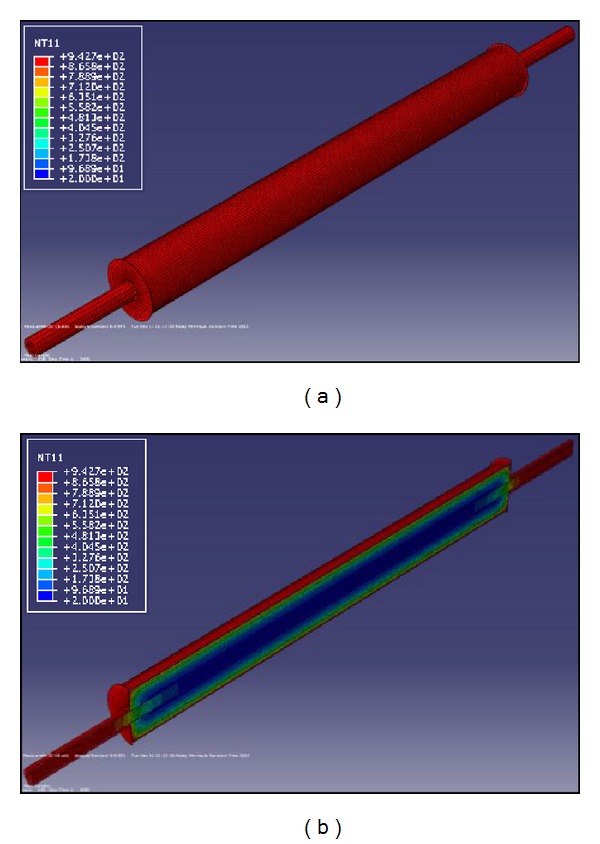
(a) Heat transfer from the fire to the exposed surface of BRB by convection and radiation, (b) heat distribution from the exterior surface of BRB to the inner surfaces.

The thermal reaction of element against heating is practically a transient heat transfer process in which the heat of fire transmits to the outer surface of the brace by convection and radiation followed by the conduction into the internal surfaces (i.e. steel tube, concrete casing, and steel core) as shown in Figure 3. Convective and radiative heat fluxes can be displayed as [11]

(1) $$\begin{array}{c} q_{\text{convection}}=h_{v}\left(T_{f}-T_{s}\right),\\ q_{\text{radiation}}=\varepsilon_{f}\varepsilon_{m}\sigma \left[{\left(T_{f}+T_{0}\right)}^{4}-{\left(T_{s}+T_{0}\right)}^{4}\right]. \end{array}$$

For standard fire exposure, the following values are proposed by EC4 [12] for composite elements: *h*_*v*_ = 25  (W/m^2^ K),  *ε*_*f*_ = 0.8, and *ε*_*m*_ = 0.7 which are used in this study. In this paper the standard ISO 834 [13] fire curve is imposed on the exterior surface of the brace as a thermal loading, so as to simulate the fire through convection and radiation heat transfer processes according to (1) (Figure 3(a)).

In the thermal analysis, the model is meshed as three-dimensional eight-node solid element (DC3D8) for steel core, concrete and steel casing. Despite a symmetrical cross-section of the brace and fire furnace on the exterior surface of the element, the whole length and entire cross-section of the brace is modeled in this study. This is because previous experiments [14] had shown that in some instances the reaction of element was governed by the shear failure of concrete. Hence unsymmetrical deformation was anticipated.

**Figure 4:**
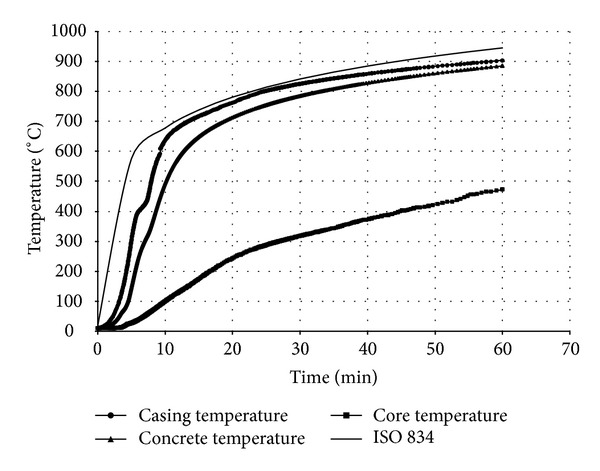
Nodal temperatures in BRB components.

**Figure 5:**
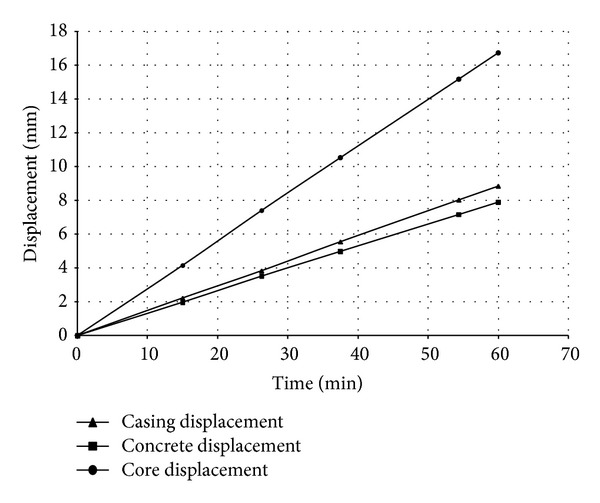
Axial displacement comparison in BRB components.

The results of the nonlinear heat transfer analysis consist of temperature-time history for the whole nodes within the 3D model. They are subsequently applied as a thermal loading to the structural model.  Figure 4 compares the nodal temperatures of BRB components as an example at the hottest position of the brace whereas Figure 5 demonstrates the corresponding nodal axial displacements.

### 2.3. Structural Analysis

**Figure 6:**
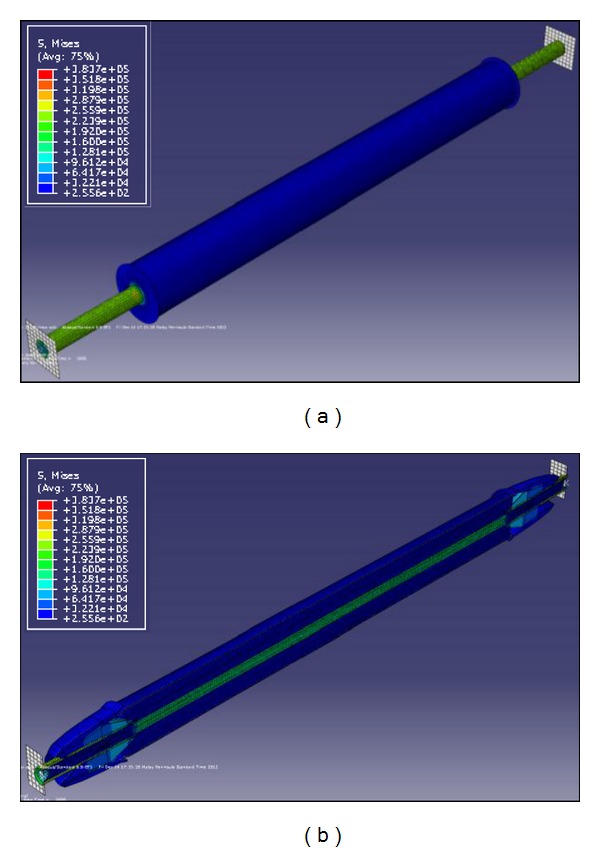
(a) 3D finite element structural model for BRBs, (b) deformed shape of BRBs after exposure to standard fire.

A nonlinear structural model is implemented after full thermal analysis, using the same finite element software [7], taking into consideration the nodal temperature-time curves previously calculated from the thermal analysis. The finite element meshes and node numberings are quite the same as those utilized in the thermal simulation. The three-dimensional eight-node solid elements (C3D8R) with reduced integration are used to mesh all parts of the bracing element. Two steel endplates are modeled in the structural analysis to implement an ending boundary condition of the steel core. The endplates are modeled as discrete rigid parts with all nodes coupled to a reference point located at the brace axis. Rigid plates are free to move along the brace axis but fixed in other translational degrees of freedom. The rigid plates are meshed using four node three-dimensional bilinear rigid quadrilateral elements (R3D4).  Figure 6 shows the three-dimensional structural model for BRB system.

As shown in Figure 6, none of the components of BRB can move freely in the axial direction. This is due to the restraint provided by two endings of bracing element. Temperature rise causes elongation in the length of the brace but as the endings of the element are restrained, a big axial force is induced in the bracing components at elevated temperature. In case of fire, it is expected that the exposed parts of the bracing member yield first. This can be seen from Figure 6(b) which shows that the most stress is experienced in the stiffeners of steel core which are added to its exposed surfaces to prevent the yielding of this part. On the other hand, it is evident that the other exposed part of BRB, that is, steel casing, has not yielded in the preliminary heating stages. It could be because in BRBs, the yield strength of steel core is intentionally established to be lower than that of steel casing with the aim of preparing the condition that the restrained part of steel core yields first, under compression force. As a direct result, a part of the brace whose strength is lower (steel core) than the other parts, yields first due to a big compression force formed in the BRB components at elevated temperature. This concept can be seen in Figure 6(b) which shows that in a fire the most axial stress is formed in the restrained part of the steel core and in the core stiffeners during primary heating stages.

### 2.4. Thermal and Mechanical Contact on Concrete-Steel Tube Casing Interface

Thermal resistance at the interface between the steel casing and in-filled concrete is modeled by applying a constant value of 200 W/m^2^K for the conductance [15]. Radiation heat transfer mechanism is ignored at the corresponding boundary because of the limited amount of air present in the interface void.

The structural interplay between the casing and in-filled concrete surfaces is modeled as follows.

1. A node-to-surface formulation is utilized in the contact approach. The normal behavior is employed as “hard contact” formulation, in which pressure transmits between the surfaces only when they have contact with each other.
2. A Coulomb friction model is used to simulate the tangential behavior of the two contact pairs. When the bond strength is higher than shear stress at the interface, no slippage between the surfaces is noted [11]. When the two contact surfaces do have relative slippage with each other, a friction or shear stress is created between them. This shear stress is determined by the coefficient of friction and the pressure in the interface. In this study, a constant coefficient of 0.3, which had formerly generated accurate results in contact between steel casing and in-filled concrete at room temperature simulation [15], has been used. The bond between the casing and the concrete is neglected, based on the consideration that bond strength may be reduced rapidly between the surfaces at high temperatures.

### 2.5. Thermal and Mechanical Contact between Concrete and Steel Core Surfaces

Thermal and mechanical characteristics of contact between the steel core and concrete are the same as those mentioned in the previous section. The only difference is that a constant tangential friction factor equal to 0.1 is used for this interface. This is because in BRB system a debonding material is applied on the surface of the core so that shear is not transferred to the restraining system. This value conforms to the friction factor between steel and rubbers.

### 2.6. Material Properties

For definition of material properties of the concrete and steel, two types of properties are needed: thermal and mechanical. For structural steel, the temperature dependent thermal properties recommended in EC3 [16] are adopted. The thermal properties of concrete at elevated temperature are taken from EC2 [17]. The moisture content of concrete is taken into account in this study, via a maximum value of specific heat, which indicates the latent heat of water vaporization. According to EC4 [12], this maximum value is 2020 J/kg K for a moisture content of 3% of concrete weight and 5600 J/kg K for moisture content of 10%. In this research, recommended value for moisture content of 3% is adopted for the siliceous aggregates in concrete.

Mechanical properties of materials in this analysis include elastic and plastic characteristics and coefficient of thermal expansion. Elastic modulus and Poisson's ratio are two principal parameters for elastic behavior. In the ABAQUS package [7], a classic metal material model is selected for steel nonlinearity which follows Von Misses' yield function and associated plastic flow rule [1]. Steel mechanical temperature-dependent values are adopted as recommended in EC3 [16].

A concrete damaged plasticity model (CDP) in ABAQUS [7] is utilized for the fundamental relationship of concrete. This model applies the significance of isotropic damaged plasticity to demonstrate the nonlinear behavior of concrete. It also contains the combination of nonassociated multihardening plasticity and isotropic damaged elasticity to demonstrate the irreversible damage which occurs through fracturing [11]. Concrete has multiple behaviors and damage mechanisms under compression and tension. Therefore, the stress-strain relationships for concrete need to be specified both in tension and compression. There are several options to define the tensile stress-strain relationship in the concrete damaged plasticity model. Tensile characteristic of concrete at elevated temperature is specified as traditional tensile stress-strain relationship that exists in the concrete damaged plasticity model as described in ABAQUS [7]. For modeling damaged plasticity in concrete, the relevant values proposed by Jankowiak [18] are adopted and presented in Table 1.

Since Euro code 3 does not provide the coefficient of thermal expansion, *α* precisely, it can be computed as the first derivative of the equations proposed for thermal strains of carbon steel, with the following result [19]:

(2.6) +20°C≤T<750°C ΔLL=1.2×10−5 T+0.4×10−8 T2 −2.41×10−4,+750°C≤T≤860°C ΔLL=1.1×10−2,+860°C<T≤1200°C ΔLL=2×10−5 T−6.2×10−3.

For concrete, the value of coefficient of thermal expansion proposed by Hong and Varma, 2009 [20], is employed.  Table 2 provides the material thermal properties for this study.

## 3. Analytical Thermal Model

The 3D numerical thermal model was validated by comparing its results with the analytical Green's function solution for transient heat conduction proposed by Wang and Tan [8]. Quick convergence of solutions at any given time interval is the main reason for using Green's function technique. It represents a preferable method to solve the transient heat conduction problems [8]. Because of this reason, this technique was used to verify the proposed numerical model results in this study. In the analytical model [8], the following assumptions were made to validate the results more conveniently.

- The outer surface of the steel casing has uniform temperature distribution with lumped capacitance behavior.
- Thermal properties of the materials are considered as constant values, that is, temperature-independent.
- The interface between steel casing and concrete is presumed to have perfect contact between surfaces.
- The length of the brace is considered to be long enough with respect to its radius. It should be exposed to the uniform heat so that the heat transfer in the longitudinal direction is eliminated.

### 3.1. Definition of the Analytical Heat Transfer Model

As mentioned previously, the whole steel casing is supposed to have a uniform temperature distribution; that is, *T*(*r*_1_ ≤ *r* ≤ *r*_2_, *t*) ≡ *T*_*s*_(*t*) and the concrete temperature at *r* = *r*_1_ is equal to *T*_*s*_(*t*). The dominant differential equation of transient heat conduction in the concrete segment is presented by [8]:

(3) $$\begin{array}{c} \frac{\partial T}{\partial t}-\frac{\alpha_{c}}{r}\frac{\partial }{\partial r}\left(r\frac{\partial T}{\partial r}\right)=0, \end{array}$$

where subscript *c* stands for concrete properties.

The natural boundary condition for convection or radiation due to fire exposure is defined as

(4) q1(t)=σε[(Tg(t)+273)4−(Ts(t)+273)4] +hc[Tg(t)−Ts(t)].

The heat flux at the interface between steel casing and the in-filled concrete is defined as

(5) $$\begin{array}{c} q_{2}\left(t\right)=q_{1}\left(t\right)-Q_{s}\frac{\partial T_{s}\left(t\right)}{\partial t}. \end{array}$$

### 3.2. Impulse Green's Function

By applying the eigenfunction concept, the impulse Green's function can be expressed as [8]:

(6) g(r,t−τ) =2αckcr0×[1+∑n=1∞1J0(ζn)e−αcζn2(t−τ)r02J0(ζnrr0)].

As the impulse Green's functions are for the homogeneous systems, some feasible singularities may occur at *t* = *τ* and *r* = *ξ* = *r*_1_ due to Dirac delta functions which represented the unit heat impulse [8]. To prevent the corresponding singularities of the impulse Green's functions *g*(*r*, *t* − *τ*), the temporary integral forms of *g*, called the step Green's functions, are used instead.

### 3.3. Step Green's Function

The step Green's function is implemented to solve the heat transfer equations as follows [8]:

(7) G(r,t)=∫τ=0tg(r,t−τ)dτ=2αctkcr0 +∑n=1∞Cn×J0(ζnrr0)(1−e−αcζn2tr02),

where the coefficient *C*_*n*_ is given by

(8) $$\begin{array}{c} C_{n}=\frac{2r_{0}}{k_{c}\zeta_{n}^{2}J_{0}\left(\zeta_{n}\right)}. \end{array}$$

### 3.4. Insertion of Fire Environment (Duhamel's Principle)

In the previous section the heat transfer model and its solution were discussed (for more details, see [8]). Duhamel's principle displays a strong technique to deal with time-dependent problems in fire conditions as [8]:

(9) $$\begin{array}{c} \theta \left(r_{1},t\right)=\theta_{s}\left(t\right)=\int_{\tau =0}^{t}q_{2}\left(t-\tau \right)dF\left(\tau \right). \end{array}$$

Let *θ*(*r*, *t*) = *T*(*r*, *t*) − *T*_0_ represent the normalized temperature field. The flux condition at the interface between the steel casing and concrete is given by

(10) $$\begin{array}{c} q_{2}\left(t\right)=q_{1}\left(t\right)-Q_{s}\dot{\theta_{s}}\left(t\right), \end{array}$$

where $\dot{\theta_{s}}(t)=\partial \theta_{s}\left(t\right)/\partial t$, with the overhead dot signifying the time derivative, and *q*_1_(*t*) is achieved at the fire exposure (with convection and conduction boundary conditions) according to (4).

For solving (9), we started with the numerical layouts by first discretizing the time interval as follows:

{*t*_*n*_ = *n*Δ*t*,  *n* = 0,1, 2,…, *N*}, where Δ*t* is the constant sampling time [8].

Consider the following:

(11) $$\begin{array}{c} \theta_{s}\left(n\right)=\int_{\tau =0}^{t}\dot{\theta_{s}}\left(\tau \right)d\tau =\left[\frac{1}{2}\dot{\theta_{s}}\left(0\right)+\sum_{j=1}^{n-1}\dot{\theta_{s}}\left(j\right)+\frac{1}{2}\dot{\theta_{s}}\left(n\right)\right]\times \Delta t\\ \dot{\theta_{s}}\left(n\right)=\frac{\left[B\left(n\right)-C\left(n\right)\right]}{A}, \end{array}$$

where

(12) A=12Δt+12QsΔF(1),B(n)=12ΔF1q1(n)+Sn(q1,ΔF),Cn=QsSn(θs˙,ΔF)+12θs˙(0)Δt+∑j=1n−1θs˙(j)Δt,Sn(υ,ΔF)=12υ(n−1)ΔF(1) +12∑j=2n[υ(n−j+1)+υ(n−j)]×ΔF(j).

In order to use the above mentioned equations perfectly, Wang and Tan [8] outlined the use of Green's function solutions step by step.

## 4. Verification of Numerical Heat Transfer Model with Analytical Thermal Model

In the analytical model [8], temperature-independent thermal properties of concrete and steel are given as

(13) $$\begin{array}{c} k_{s}=45\;\text{W}/\text{mk},\;\;C_{s}=600\;\text{J}/\text{kg}\;\text{K},\;\;\rho_{s}=7850\;\text{kg}/{\text{m}}^{3},\\ k_{c}=1.6\;\text{W}/\text{mk},\;\;C_{c}=1000\;\text{J}/\text{kg}\;\text{K},\;\;\rho_{c}=2400\;\text{kg}/{\text{m}}^{3}. \end{array}$$

**Figure 7:**
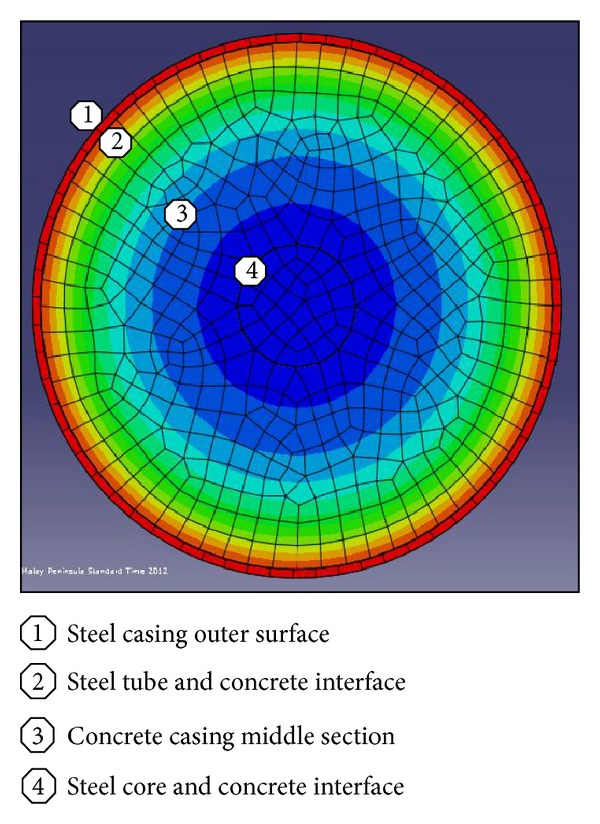
Different locations of bracing elements for temperature predictions.

**Figure 8:**
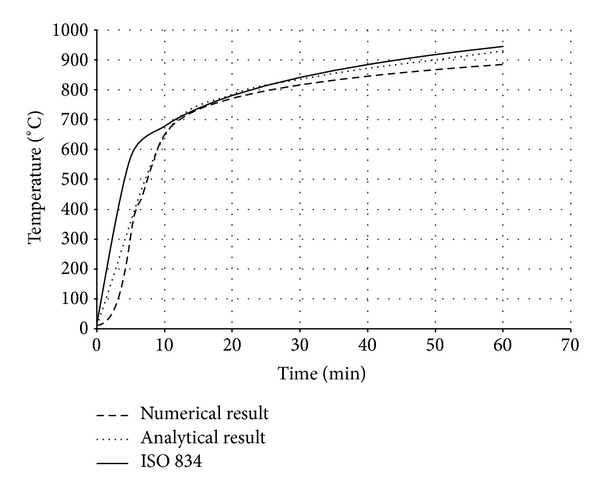
Comparison of *T*-*t* Curves at point 1.

**Figure 9:**
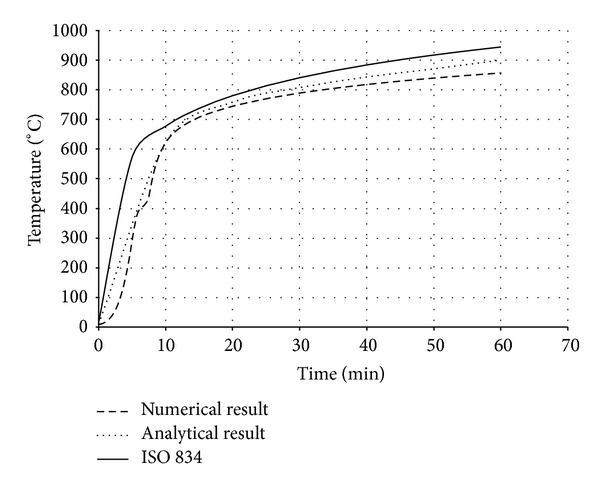
Comparison of *T*-*t* Curves at point 2.

**Figure 10:**
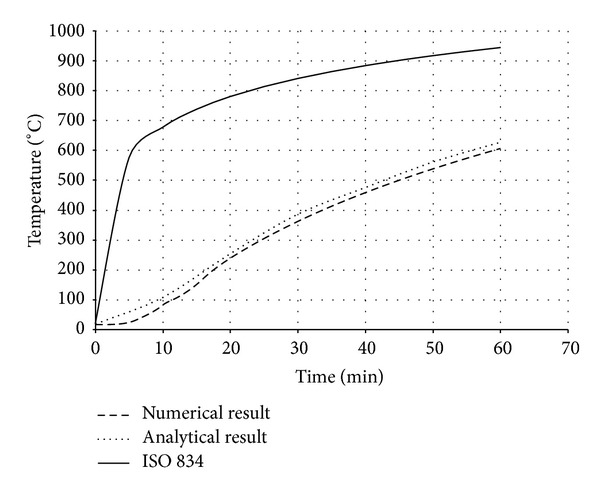
Comparison of *T*-*t* Curves at point 3.

**Figure 11:**
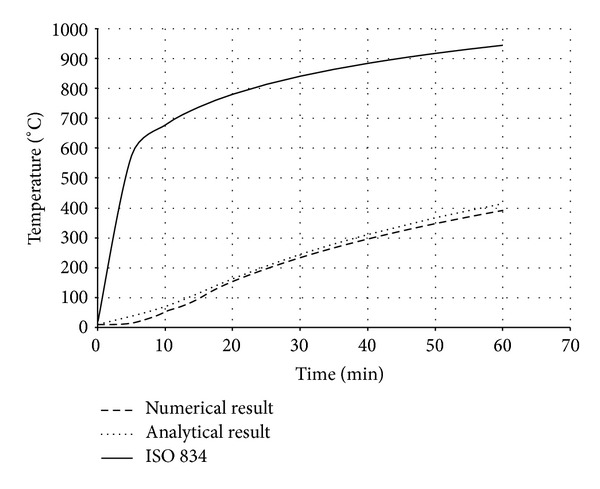
Comparison of *T*-*t* Curves at point 4.

Also in the analytical model, the existence of moisture content in concrete part was not taken into consideration for simplicity. The temperature-time responses (*T*-*t* Curves) at different locations of bracing section (Figure 7) are compared in both analysis procedures (Figures 8, 9, 10, and 11). The values in these figures show that the temperature-time histories, derived from numerical model in every location of BRB section, are in good agreement with the analytical solution prediction. Some discrepancies between the results are predicted by the analytical equations [8] and the proposed FE analysis. This could be due to some previously mentioned assumptions that were considered in the analytical solution for simplicity.

It is worth mentioning here that the results of the proposed FE model are in close agreement with the experimental work on the behavior of BRB with square cross section in case of fire. This work was carried out by Saitoh et al., 2005 [21].

## 5. Analytical Structural Model 

### 5.1. Theory and Assumption

The proposed FE model for the structural behavior of BRB under axial force due to temperature rise was validated by the analytical model proposed by Choi and Xiao [9]. This study predicted lateral interaction between concrete and steel casing in compression. In the analytical model [9], it was assumed that each segment supported the stress only from the axial direction with no stress from the other directions. Furthermore, the concrete surface and the steel casing internal surface have a complete contact between them at room temperature; that is, there is no gap between the concrete and tube at room temperature. At elevated temperature; on the other hand, the cross-sectional interaction between them is such that the gap is developed between steel casing and in-filled concrete during axial thermal loading. This is because the coefficient of thermal expansion of concrete is less than steel. Hence, the steel casing expands more than concrete laterally and creates a gap at the interface. In this state, the steel casing and in-filled concrete deformations are similar as in uniaxial loading of each specimen. So both materials will be deformed without any interaction. As mentioned earlier that in BRB there is an initial gap between the outer surface of the core and the concrete; thus the steel core deformation is also in uniaxial loading mode and without any interaction with the concrete. It is worth mentioning here that the bond stress at the steel casing and in-filled concrete interface is assumed to be zero.

### 5.2. Equilibrium Condition

**Figure 12:**
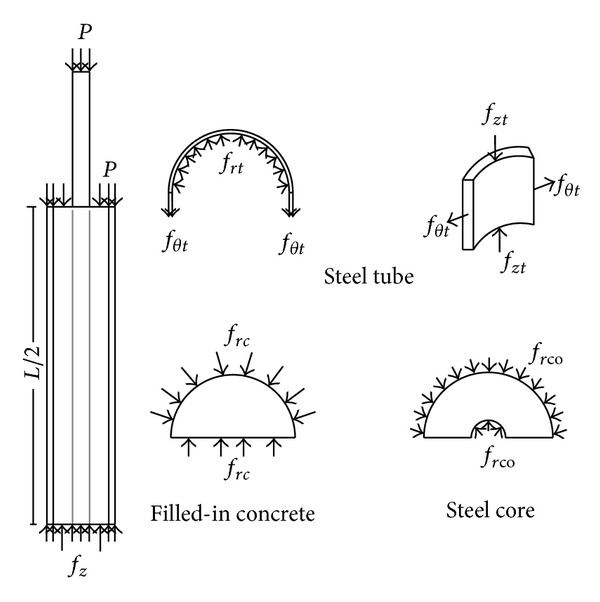
Equilibrium relationship between BRB components.

Figure 12 displays the cylindrical coordinate system of the BRB system in the equilibrium state. In the equilibrium equations, the *r*-direction and *θ*-direction stresses of the steel core and the concrete are equal. As per relationship between steel tube and concrete, the equilibrium conditions can be described by the following [9]:

(14) $$\begin{array}{c} f_{rc}=f_{\theta c},\\ 2t_{t}\times f_{\theta t}=-f_{rc}\left(D_{t}-2t_{t}\right), \end{array}$$

where subscripts *c*, *t*, and co denote concrete, steel tube, and core, respectively. At elevated temperatures, the axial compression force in the bracing element can be represented as

(15) $$\begin{array}{c} P_{\text{th}}=EA\alpha \cdot \Delta \theta . \end{array}$$

In case the gap between steel tube and concrete interface does not exist at room temperature, *θ*-direction strain values should be the same between them. Also the *r*-direction and *θ*-direction strains of concrete and core remain identical. Hence, the deformation relationships can be expressed as [9]:

(16) $$\begin{array}{c} r\text{-}\theta \;\;\text{direction:}\;\varepsilon_{rc}=\varepsilon_{\theta c},\\ r\text{-}\theta \;\;\text{direction:}\;\varepsilon_{r\mathrm{co}}=\varepsilon_{\theta \mathrm{co}},\\ \theta \;\;\text{direction:}\;\varepsilon_{\theta c}=\varepsilon_{\theta t}. \end{array}$$

### 5.3. Constitutive Model

#### 5.3.1. Constitutive Model for Steel Tube Casing

In the steel casing, the stress-strain relationship can be displayed by Hooke's law under biaxial stress as [9]:

(17) $$\begin{array}{c} \left\{\begin{array}{c} f_{zt}\\ f_{\theta t} \end{array}\right\}=\frac{E_{s}}{1-\upsilon_{t}^{2}}\left[\begin{array}{cc} 1 & \nu_{t}\\ \nu_{t} & 1 \end{array}\right]\left\{\begin{array}{c} \varepsilon_{zt}\\ \varepsilon_{\theta t} \end{array}\right\}, \end{array}$$

where *f*_*zt*_ and *f*_*θt*_ are axial and tangential forces, and *ε*_*zt*_ and *ε*_*θt*_ represent axial and tangential strains in the steel tube casing, respectively.

#### 5.3.2. Constitutive Model for Concrete

The concrete model is based on octahedral normal and shear stress-strain relationships, which can be defined by the following [9]:

(18) $$\begin{array}{c} \sigma_{\text{oct}}=\frac{1}{3}\left(f_{z}+f_{r}+f_{\theta }\right),\\ \tau_{\text{oct}}=\frac{1}{3}\sqrt{{\left(f_{z}-f_{r}\right)}^{2}+{\left(f_{r}-f_{\theta }\right)}^{2}+{\left(f_{\theta }-f_{z}\right)}^{2}},\\ \varepsilon_{\text{oct}}=\frac{1}{3}\left(\varepsilon_{z}+\varepsilon_{r}+\varepsilon_{\theta }\right),\\ \gamma_{\text{oct}}=\frac{2}{3}\sqrt{{\left(\varepsilon_{z}-\varepsilon_{r}\right)}^{2}+{\left(\varepsilon_{r}-\varepsilon_{\theta }\right)}^{2}+{\left(\varepsilon_{\theta }-\varepsilon_{z}\right)}^{2}}, \end{array}$$

where *σ*_oct_ and *τ*_oct_ are octahedral normal and shear stresses and *ε*_oct_ and *γ*_oct_ are octahedral normal and shear strains, respectively.

#### 5.3.3. Constitutive Model for Steel Core

The stress-strain relationship of steel core for the elastic range can be represented by the following Hooke's law under three-axial stress:

(19) {fzcofrcofθco}=Ec(1+υco)(1−2υco) ×[1−υcoυcoυcoυco1−υcoυcoυcoυco1−υco]{εzcoεrcoεθco},

where *f*_*z*co_, *f*_*r*co_, and *f*_*θ*co_ are axial, radial and tangential stresses and *ε*_*z*co_, *ε*_*r*co_, and *ε*_*θ*co_ are axial, radial, and tangential strains in the steel core, respectively.

## 6. Verification of Numerical Structural Analysis with Analytical Model

**Figure 13:**
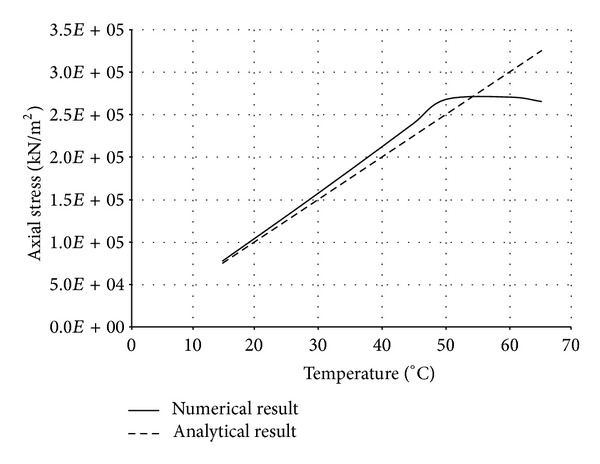
Comparison of axial stress in steel core.

**Figure 14:**
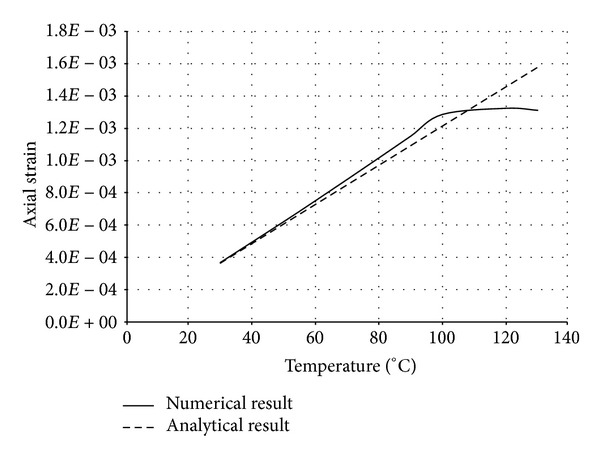
Comparison of axial strain in steel core.

**Figure 15:**
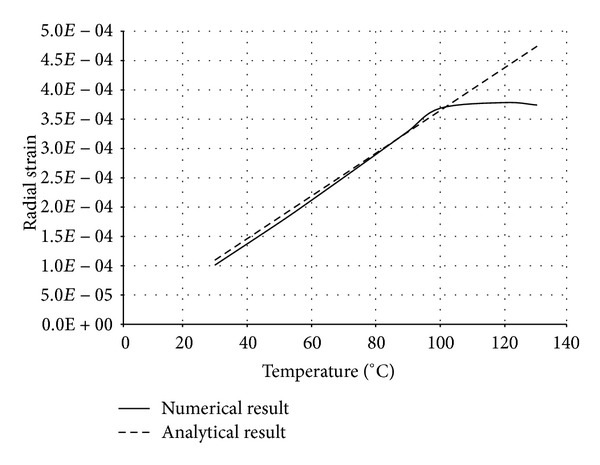
Comparison of radial strain in steel core.

**Figure 16:**
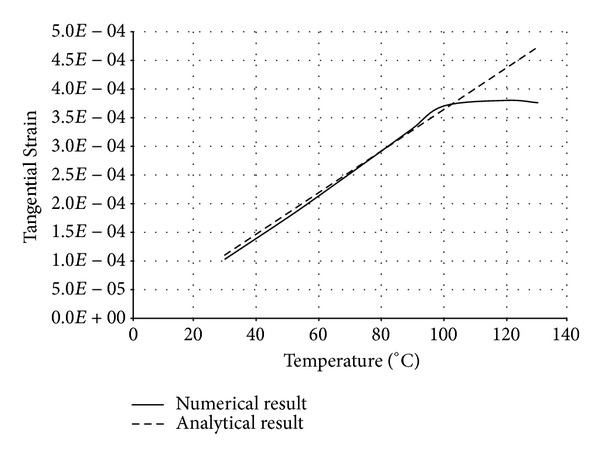
Comparison of tangential strain in steel core.

**Figure 17:**
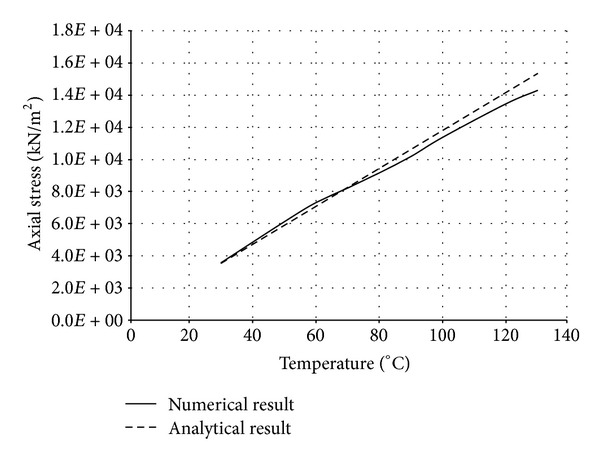
Comparison of axial stress in concrete.

**Figure 18:**
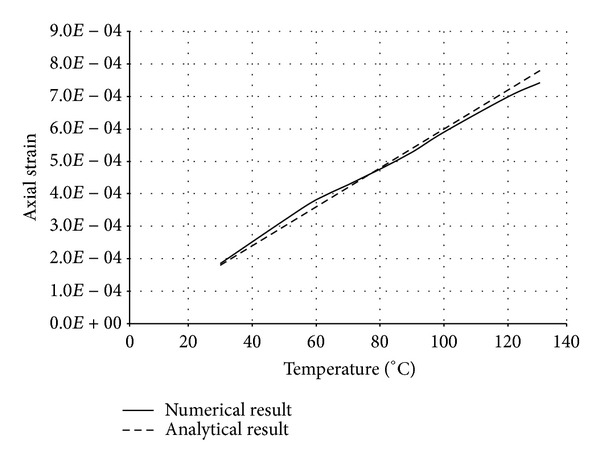
Comparison of axial strain in concrete.

**Figure 19:**
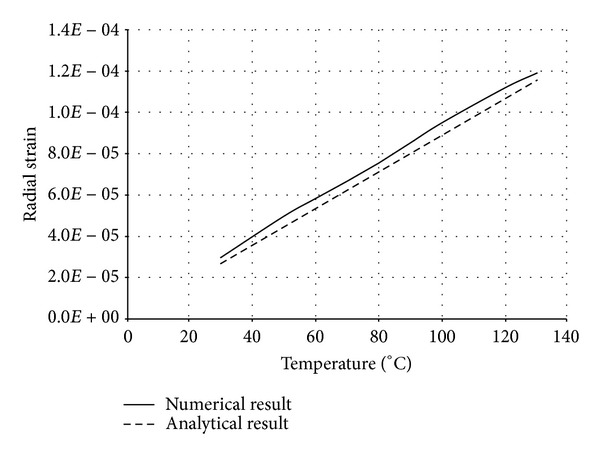
Comparison of radial strain in concrete.

**Figure 20:**
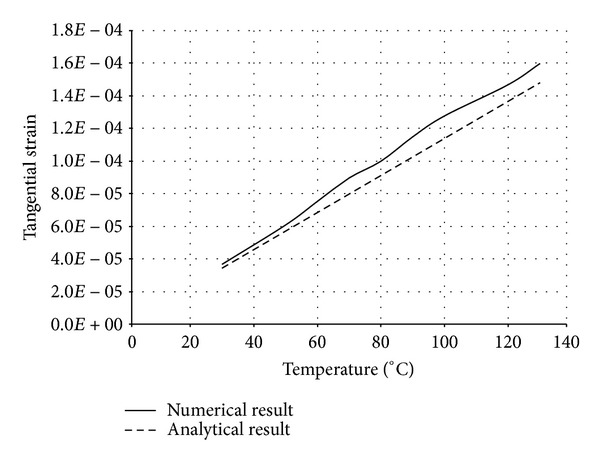
Comparison of tangential strain in concrete.

**Figure 21:**
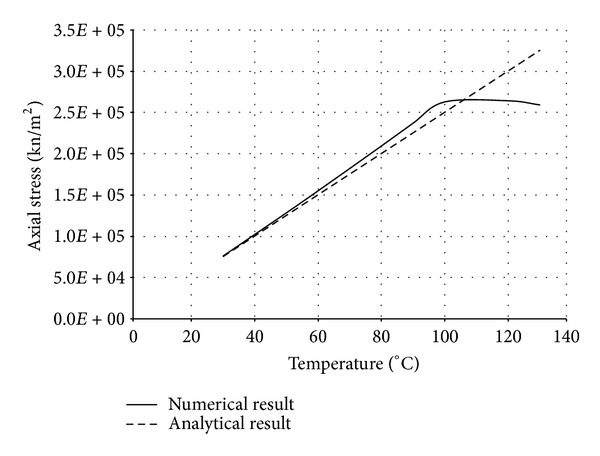
Comparison of axial stress in steel tube casing.

**Figure 22:**
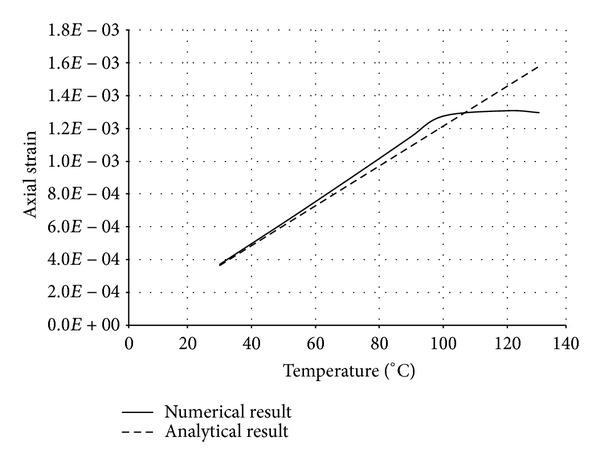
Comparison of axial strain in steel tube casing.

**Figure 23:**
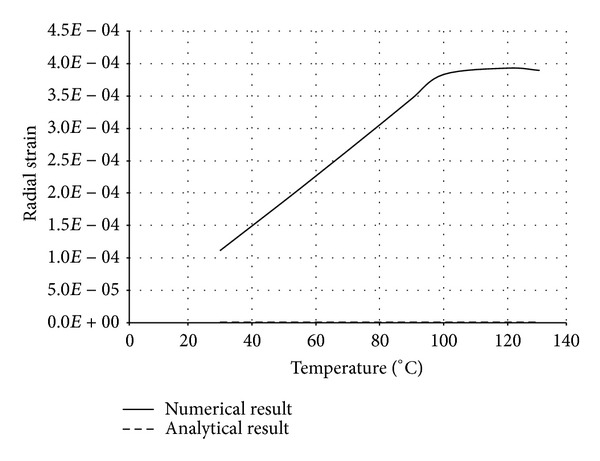
Comparison of radial strain in steel casing.

**Figure 24:**
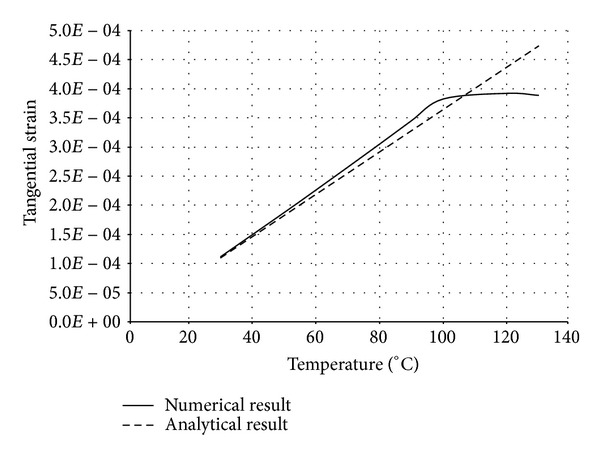
Comparison of tangential strain in steel casing.

The numerical structural model was validated by comparing the results with the analytical solution for composite elements subjected to axial compression force, as proposed by Choi and Xiao [9]. In this comparison, the elastic range of materials is discussed with an initial gap between the steel core and concrete as well as a gap generated between them during loading at elevated temperatures. The adequacy of the numerical results developed by the authors was verified by comparing with the results of existing analytical structural formulations [9] (Figures 13–24).

### 6.1. Verification of Core Results

In the analytical simulation, the stress in *r*-*θ* direction was set to zero. This is because of the existence of initial gap between steel core and concrete in the BRB system. Figures 13, 14, 15, and 16 compare the stresses and strains, resulting in both analytical and proposed numerical models.

### 6.2. Verification of Filled-In Concrete Results

Because of the generated gap between steel casing and concrete at elevated temperature, the radial and tangential stresses between them in the restraining system were set to zero value in the analytical model. Figures 17, 18, 19, and 20 compare the stress and strain results in both procedures. The data showed that the proposed numerical FE model results are generally in good agreement with the analytical results.

### 6.3. Verification of Steel Casing Results in Restraining System

Figures 21, 22, 23, and 24 compare the stress and strain curves for both procedures in the steel tube casing. Close agreement with the analytical and numerical results in the behavior of steel casing at elevated temperature was observed. In the steel tube, the radial strain value is near zero, so its thickness is negligible in comparison to its other dimensions. Thus in the analytical simulation radial strain was set to zero. This is consistent with the low numerical values shown in Figure 23.

The comparisons between the numerical and analytical results showed that the proposed FE analysis of Buckling Restrained Brace is relatively close to the analytical results, as illustrated in Figures 13–24. According to the yield criteria proposed by Choi and Xiao [9], concrete and steel casing behave linearly till temperatures of 225°C and 196°C, respectively. This result was also observed in our FE analysis, which leads to the nonlinear behavior of materials in the restraining system of BRB, at temperatures higher than these values. As the aim of this comparison was to validate the linear behavior of materials in BRB, the concrete temperatures below 225°C and steel tube temperatures below 196°C were applied in the analytical simulation.

## 7. Conclusions

The numerical model consisting of two sequentially coupled analysis steps, namely, heat transfer and stress was utilized to represent the behavior of BRB exposed to standard fire loading. Proposed FE models were verified at each stage with the analytical approaches. The analytical methods based on Green's function solution proposed by Wang and Tan [8] and analytical studies on composite elements structural behavior, proposed by Choi and Xiao [9] were selected to verify the ability of numerical heat transfer and structural analyses, respectively.

It should be noted that structural verifications with analytical results were limited to the linear behavior of bracing element components, at elevated temperature. Therefore, research work reported in this paper provides reference for further nonlinear structural verifications of BRBs exposed to elevated temperature or fire. Results and discussions lead us to the following conclusions.

- The behavior of BRB has been studied numerically when exposed to fire from all sides. The FE findings have showed that failure of bracing element occurs as a result of both reduction in material strength and increase compression stresses at elevated temperatures.
- The surface temperature of BRB system increased with the rise in environmental temperature and conducted through the inner surface of element.
- The numerical thermal results reflect the temperature change of component section clearly and intuitively.
  - The temperature gradient was reduced gradually along the normal direction of steel tube surface suffering fire.
  - The outside surface had the highest temperature and the temperature gradient of far section, that is, the steel core section at middle length of the brace was the coolest area.
- The FE predicted temperatures and stress-strain relations correspond to the analytical results.
- The limiting temperatures in the linear behavior of steel casing and concrete in the analytical simulation were 196°C and 225°C, respectively. These results also supported the FE analysis, which leads to the nonlinear behavior of materials in the restraining system of BRB, at temperatures higher than these values.
- In incidents of fire caused by earthquakes, since the steel core suffers both axial seismic and thermal forces, and steel casing just thermal force in BRB, the axial compression force in the former will be more than latter. On the other hand, because the yield strength of core is less than casing, the formation of first plastic hinges will occur in the steel core. Thus the core yields prior to the casing. It appeared that the behavior of BRB at elevated temperatures turned out to be the same as at room temperature.
- In situations of fire (without the axial seismic force), although the whole part of the casing was exposed to fire and the expansion of tube was more than restraint part of the core, our findings on FE results show that the yield of core occurred prior to the casing. This is because the yield strength of the former is much lower than the latter. Hence, the rate of yielding in the core is much higher than the expansion of the casing due to the creation of a big axial force within it at high temperatures. In this case again it was seen that the behavior of BRB at elevated temperature is the same as at room temperature.
- Based on this research study, it is expected that BRBs manifest excellent structural behavior against fire hazards as well as earthquakes due to their symmetrical response in tension and compression.
- This research study provided a theoretical basis for understanding further high-temperature structural properties and refractory limit of BRBs exposed to fire.

## Figures and Tables

**Table 1:** Material parameters of CDP model for concrete [18].

Concrete elasticity		Parameters of CDP model	
		*β*	38°
		*m*	1
*E* (Gpa)	19.7	*f* = *f*_*b*0_/*f*_*c*_	1.12
*ν*	0.19	*γ*	0.67

Concrete compression hardening		Concrete compression damage	
Stress (Mpa)	Crushing strain	Damage	Crushing strain

15.00	0.000000	0.000000	0.000000
20.20	0.000075	0.000000	0.000074
30.00	0.000100	0.000000	0.000099
40.30	0.000154	0.000000	0.000154
50.01	0.000761	0.000000	0.000761
40.24	0.002557	0.195402	0.002557
20.24	0.005675	0.596382	0.005675
5.26	0.011733	0.894865	0.011733

Concrete tension stiffening		Concrete tension damage	
Stress (MPa)	Cracking strain	Damage	Cracking strain

1.20	0.000000	0.000000	0.000000
2.84	0.000030	0.000000	0.000033
1.87	0.000160	0.406411	0.000160
0.86	0.000279	0.696380	0.000279
0.23	0.000684	0.920389	0.000684
0.06	0.001087	0.980093	0.001086

**Table 2:** Thermal properties of steel and concrete at elevated temperature.

Temperature	Thermal expansion (×10^−6^)		Thermal conductivity (J/S-m-°C)		Specific Heat (×10^−2^)	
	Concrete	Steel	Concrete	Steel	Concrete	Steel
20	6.00	12.16	1.45	53.30	9.00	4.40
100	6.80	12.80	1.35	50.70	9.00	4.88
200	7.60	13.60	1.33	47.30	20.20	5.30
300	8.40	14.40	1.22	44.00	10.00	5.65
400	9.20	15.20	1.10	40.70	10.50	6.06
410	9.30	16.00	0.99	37.40	11.00	6.67
445	9.60	16.80	0.89	34.00	11.00	7.60
500	10.00	17.60	0.80	30.70	11.00	10.08
600	10.80	0.00	0.72	29.50	11.00	50.00
635	11.10	20.00	0.67	27.30	11.00	8.03
700	11.60	20.00	0.63	27.30	11.00	6.50
715	11.70	20.00	0.60	27.30	11.00	6.50
785	12.30	20.00	0.57	27.30	11.00	6.50
800	12.40	12.16	0.55	27.30	11.00	6.50
900	13.20	12.80	1.45	53.30	9.00	4.40
1000	14.00	13.60	1.35	50.70	9.00	4.88
1100	14.80	14.40	1.33	47.30	20.20	5.30
1200	15.60	15.20	1.22	44.00	10.00	5.65

## Notations

*q*: Heat flux
*T*_*f*_: Temperature of fire
*T*_*s*_: Outer steel casing temperature
*T*_0_: Absolute zero temperature
*h*_*v*_: Heat convective coefficient
*ε*_*f*_: Emissivity of fire
*ε*_*m*_: Emissivity of the steel surface
*σ*: Stefan Boltzmann constant
*β*: Dilation angle
*f* = *f*_*b*0_/*f*_*c*_: The parameter is defined by the Kupfer's curve
*f*_*b*0_: Compressive strength under biaxial loading of concrete
*m*: Eccentricity of the plastic potential surface
*ν*: Poison's ratio
*r*_0_: Steel core cylindrical radius
*r*_1_: Concrete cylindrical radius
*r*_2_: Steel tube casing
*α* = *k*/*ρc*: Thermal diffusivity
*k*: Thermal conductivity
*c*: Specific heat
*ρ*: Density
*Jn*(*z*): Bessel function of the first kind of order *n*
*ζ*_*n*_: Positive roots of *J*_1_(*ζ*_*n*_) = 0
*f*_*r*_: Radial force
*f*_*θ*_: Tangential force
*f*_*z*_: Axial force
*t*_*t*_: Tube thickness
*D*_*t*_: Tube diameter
Δ*L*: Thermal elongation
*P*: Thermal force
*ϵ*_*r*_: Radial strain
*ϵ*_*θ*_: Tangential strain
*ϵ*_*z*_: Axial strain
*σ*_oct_: Octahedral normal stress
*τ*_oct_: Octahedral shear stress
*ϵ*_oct_: Octahedral normal strain
*γ*_oct_: Octahedral shear strain
*K*: Bulk modulus
*G*: Shear modulus
*f*_*bc*_ = *f*_*c*_: Compressive strength of concrete
*f*_*y*_: Yield strength of steel
*Q*_*s*_(∂*T*_*s*_(*t*)/∂*t*): Heat storage inside the steel section
*q*_1_(*t*): Heat flux entering the entire element (BRB)
*q*_2_(*t*): Heat flux leaving the steel section and entering the concrete section.
